# Photochemical Activity of Black Phosphorus for Near‐Infrared Light Controlled In Situ Biomineralization

**DOI:** 10.1002/advs.202000439

**Published:** 2020-05-27

**Authors:** Jundong Shao, Changshun Ruan, Hanhan Xie, Paul K. Chu, Xue‐Feng Yu

**Affiliations:** ^1^ Materials and Interfaces Center Shenzhen Institutes of Advanced Technology Chinese Academy of Sciences Shenzhen 518055 P. R. China; ^2^ International Cancer Center Laboratory of Evolutionary Theranostics (LET) School of Biomedical Engineering Health Science Center Shenzhen University Shenzhen 518060 P. R. China; ^3^ Department of Physics Department of Materials Science and Engineering and Department of Biomedical Engineering City University of Hong Kong Tat Chee Avenue Kowloon Hong Kong 999077 China

**Keywords:** biodegradability, biomineralization, black phosphorus, near‐infrared light, 2D materials

## Abstract

The photochemical activity of black phosphorus (BP) in near‐infrared (NIR) light controlled in situ biomineralization is investigated. Owing to the excellent NIR absorption, irradiation with NIR light not only promotes degradation of BP into PO_4_^3−^, but also enhances the chemical activity to accelerate the reaction between PO_4_^3−^ and Ca^2+^ and promote in situ biomineralization. Mineralization of hydrogels is demonstrated by the preparation of BP incorporated hydrogel (BP@Hydrogel) which delivers greatly improved biomineralization performance under NIR illumination. The biomineralization process which can be controlled by modulating the light irradiation time and location has a high potential in controlling the mechanical properties and osteoinductive ability in tissue engineering. This study also provides insights into the degradation, photochemical activity, and new biological/biomedical applications of BP.

## Graphical abstract

Photochemical activity of black phosphorus (BP) for controlled in situ biomineralization is investigated. Near infrared (NIR) light can promote the degradation of BP and enhance their chemical activity to accelerate the mineralization process. BP@Hydrogel with NIR irradiation exhibit greatly improved biomineralization and can be controlled by modulating the irradiation time and location, thus promising high potential in bioengineering.

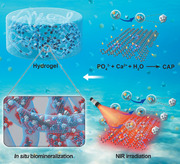

## 1 Introduction

As a new and emerging 2D semiconductor containing a single element of phosphorus, black phosphorus (BP) has been shown to have superior physical properties and great potential in a myriad of applications including optoelectronics, catalysis, energy, and biomedicine.^[^ ^1^, ^2^, ^3^, ^4^, ^5^, ^6^, ^7^, ^8^ ^]^ Particularly, few‐layer BP sheets also called phosphorene have excellent optical properties, such as the layer‐dependent bandgap, broad optical absorption range spanning the near‐infrared (NIR) region, as well as high photothermal and photodynamic efficiency^[^ ^9^, ^10^, ^11^, ^12^, ^13^ ^]^ Moreover, owing to the good biocompatibility and biodegradability, BP sheets have captured extensive attention in biomedical applications, such as optical therapies, drug/gene delivery, bioimaging, and sensing.^[^ ^14^, ^15^, ^16^, ^17^, ^18^, ^19^, ^20^, ^21^, ^22^ ^]^ However, in spite of the excellent properties, BP sheets degrade rapidly in the presence of oxygen and/or water and degradation is accelerated by light irradiation^[^ ^23^, ^24^, ^25^, ^26^ ^]^ thus hindering many potential applications.^[^ ^27^, ^28^, ^29^ ^]^ However, the degradable nature of BP sheets can be exploited to boost the chemical activity. Typically, degradation of BP in the presence of water produces phosphate (PO_4_^3−^),^[^ ^30^, ^31^, ^32^ ^]^ which is a vital constituent in bone mineralization, phospholipids in membranes, nucleotides that provide energy and found in DNA and RNA, and phosphorylated intermediates in cellular signaling. Phosphate thus plays a critical role in skeletal development, mineral metabolism, and cellular functions in the human body.^[^ ^33^, ^34^, ^35^ ^]^ Although the degradation‐induced chemical activity of BP sheets and concomitant photoresponsivity are very interesting, there have been few systematic investigations and the related phenomena are not well understood.

Herein, the chemical and photochemical activity of BP is studied systematically and the potential use in NIR‐light controlled in situ biomineralization is investigated. PO_4_^3−^ combines with calcium (Ca^2+^) in the physiological environment to form the calcium phosphate (CAP) biomineral found in bone, teeth, and tendons.^[^ ^36^, ^37^, ^38^ ^]^ In fact, the phosphorus‐driven and calcium‐extracted biomineralization processes have immense potential in many applications, such as particle reinforcement of biomaterials, biomimetic materials, and tissue engineering.^[^ ^39^, ^40^, ^41^ ^]^ Typically, biomineralization is implemented to adjust the mechanical properties of hydrogels via particle reinforcement of calcium phosphate nanostructures and can also be applied in vivo to generate implants with tunable mechanical properties.^[^ ^42^, ^43^, ^44^ ^]^ In addition, biomineralization plays a crucial role in the growth of hard tissues, such as teeth and bone and also the initiation of bone regeneration.^[^ ^40^ ^]^ Generally, biomineralization begins with a precipitation reaction from different ions (such as Ca^2+^, CO_3_^2−^, and PO_4_^3−^) followed by nucleation, growth, and self‐organization of the mineral crystals, which is controlled by matrix macromolecules in the living organisms.^[^ ^45^ ^]^ The biomineralization process usually proceeds spontaneously in organisms and it is difficult to control the process artificially in situ in the body.

**Scheme 1:**
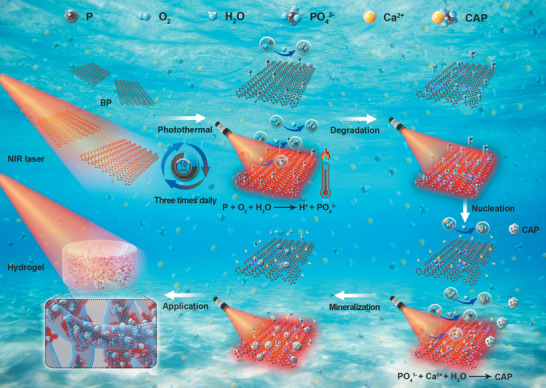
Schematic illustration of the NIR photochemical activity of BP to control the biomineralization of hydrogel in situ.

Our results demonstrate that BP enables controllable in situ formation of PO_4_^3−^ for the biomineralization of hydrogels (**Scheme** **1**). Owing to the excellent NIR absorption properties of BP sheets, irradiation with NIR light not only promotes degradation of BP into PO_4_^3−^ but also enhances the chemical activity to accelerate the reaction between PO_4_^3−^ and Ca^2+^ to promote in situ biomineralization. This process is demonstrated by the mineralization of hydrogels to improve the mechanical properties and in vivo biomineralization induction ability. Furthermore, owing to the high tissue penetration ability and excellent controllability in space and time of NIR light, the NIR light‐induced photochemical activity of BP provides an efficient way to spatially tune the mineralization behavior by modulating the irradiation time and location.

## 2 Results and Discussion

### 2.1 Synthesis and Characterization of BP Sheets

**Figure 1:**
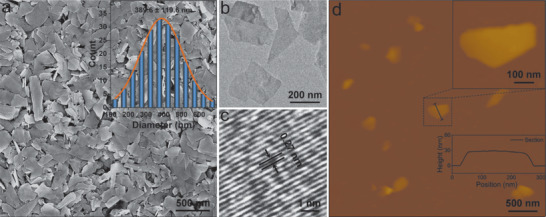
Characterization of BP sheets: a) SEM image and statistical size analysis (inset). b) TEM image. c) HR‐TEM image. d) AFM images and cross‐sectional analysis (inset) of BP sheets.

The BP sheets are synthesized by a modified liquid exfoliation technique reported by our group previously.^[^ ^16^ ^]^ The scanning electron microscopy (SEM) image in **Figure** **1**a reveals a uniform morphology with an average lateral size of 389.6 ± 119.6 nm (inset in Figure 1a) according to the statistical analysis of 200 BP sheets. The transmission electron microscopy (TEM) image in Figure 1b and high‐resolution TEM (HR‐TEM) image in Figure 1c discloses lattice fringes of 0.27 nm matching that of the monolayered BP structure. The atomic force microscopy (AFM) image in Figure 1d shows the topographic morphology of the BP sheets and the thickness is determined by the cross‐sectional analysis (inset in Figure 1d). A 2D sheet‐like morphology with a flat and smooth surface is clearly observed and the average thickness of the BP sheets is 27.1 ± 9.3 nm. Raman spectrum in Figure S1 (Supporting Information) reveals three prominent Raman peaks at 355.4, 430.8, and 459.3 cm^−1^, which can be assigned to one out‐of‐plane phonon mode (A^1^_g_) and two in‐plane modes (B_2g_ and A^2^_g_), respectively.^[^ ^16^ ^]^ To evaluate the NIR photothermal performance, 20 ppm BP sheets dispersed in aqueous solution was exposed to an NIR laser (808 nm, 1.0 W cm^−2^) for 10 min. As shown in Figure S2 (Supporting Information), the solution temperature increases by 26.1 °C after irradiation, while the temperature of water increases by only 2.4 °C, indicating the excellent photothermal performance of BP sheets.

### 2.2 The Degradation Process of BP Sheets

**Figure 2:**
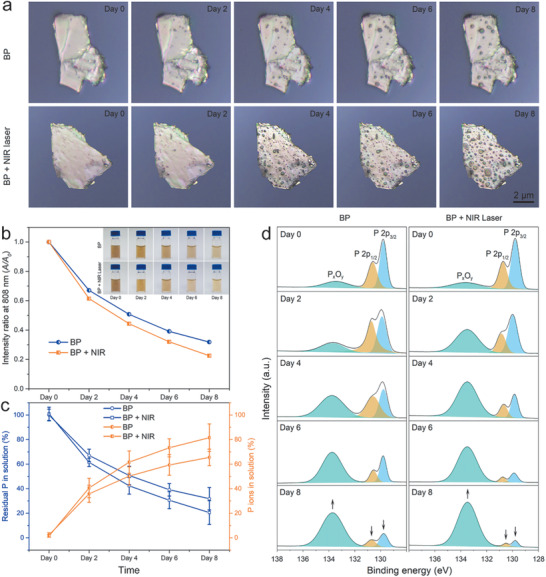
Degradation process of BP sheets without/with NIR laser irradiation: a) Photographs of a BP sheet without/with NIR light irradiation in moist air for 0, 2, 4, 6, and 8 days. b) Variation of the absorption ratios (*A*/*A*_0_) at 808 nm and corresponding photographs of BP sheets in the solution without/with NIR laser irradiation for 0, 2, 4, 6, and 8 days. c) Quantitative analysis of the residual P and P ions in the solution of the BP sheets without/with NIR laser irradiation after storing in water for different periods of time. d) P 2p XPS spectra of BP sheets after storing in water for different periods of time.

The degradation properties of BP sheets are studied. As shown in **Figure** **2**a, when a big BP sheet is exposed to nearly 100% relative humidity air at room temperature for 2 days, small topographic protrusions (hereafter termed “bubbles”) emerge from the surface and both the density and size of the bubbles increase with time as a result of structural or chemical changes.^[^ ^46^ ^]^ When the BP sheet in the same environment is exposed to 808 nm light (1.0 W cm^−2^, 10 min) three times daily, their surface becomes rougher compared to that without light irradiation for the same exposure time (Figure 2a) suggesting that the chemical activity of BP increases with NIR light illumination.

To further evaluate the degradation process, the aqueous dispersions containing the same amount of BP sheets (20 ppm) are exposed to air for 8 days without or with NIR light irradiation (1.0 W cm^−2^, 10 min, three times daily). The absorption spectra and variation of the absorption ratios (*A*/*A*_0_) at 808 nm in Figure S3 (Supporting Information); and Figure 2b shows that the BP sheets with NIR light irradiation show faster degradation as demonstrated by the faster decline of the absorbance intensity compared to that without NIR light irradiation. The corresponding photographs in the inset of Figure 2b show that the color of these two dispersions becomes lighter and the dispersion with NIR light irradiation shows more severe color deterioration than that without irradiation after 2, 4, 6, and 8 days. The amount of residual BP and the concentration of P ions in the solution are determined by inductively‐coupled plasma atomic emission spectroscopy (ICP‐AES) (Figure 2c). The BP sheets under NIR light irradiation degrades nearly 30% faster than without NIR light irradiation after 8 days and the trend of the absorbance intensity is similar.

Since degradation of BP is caused by the reaction with oxygen and water to form oxidized phosphorus species (P*_x_*O*_y_*) followed by the subsequent reaction of P*_x_*O*_y_* to PO_4_^3−^ ions,^[^ ^30^ ^]^ X‐ray photoelectron spectroscopy (XPS) is employed to determine the chemical states of the BP sheets without/with NIR light irradiation during degradation. As shown in the P 2p spectra in Figure 2d, the two peaks at 129.5 and 130.5 eV verify the original state of BP and that at 134.0 eV is associated with P*_x_*O*_y_*.^[^ ^47^, ^48^, ^49^, ^50^ ^]^ After NIR light irradiation, the P*_x_*O*_y_* peak increases more than that without NIR light irradiation, indicating more serious oxidization of BP sheets under NIR illumination. The concentration of PO_4_^3−^ after degradation is determined with the phosphate assay kit and the trend is similar to that disclosed by XPS that the BP sheets with NIR light irradiation produce more PO_4_^3−^ in the supernatant (Figure S4, Supporting Information). The results indicate the enhanced photochemical activity of BP by NIR illumination accelerates degradation and PO_4_^3−^ a production that can facilitate biomineralization.

### 2.3 In Situ Biomineralization of BP Sheets

**Figure 3:**
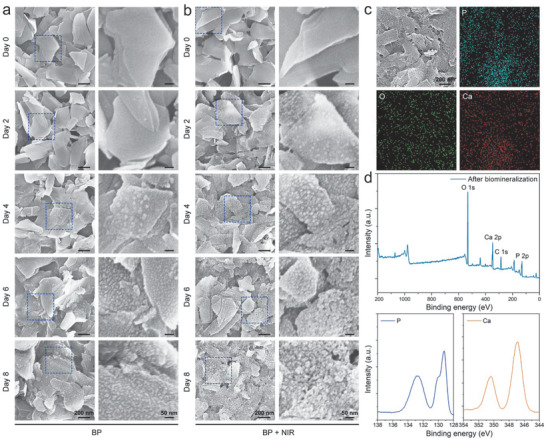
Characterization of BP sheets after biomineralization: SEM images of BP sheets a) without and b) with NIR light irradiation after biomineralization in SBF for different periods of time. c) EDS elemental maps and d) XPS spectra of BP sheets after biomineralization in SBF for 8 days.

The in situ biomineralization properties of BP sheets are determined in a simulated physiological environment by dispersing them in a simulated body fluid (SBF) and shaking at 37 °C. SBF is a suitable medium to study the biomineralization performance of BP sheets in vitro.^[^ ^51^ ^]^ Compared to BP quantum dots (≈5 nm) and BP bulks (over 10 µm), BP sheets exhibit proper degradation rate in SBF, which is more suitable for long‐term biomineralization (Figure S5, Supporting Information). After 2, 4, 6, and 8 days, the BP sheets are removed from the SBF and washed gently with distilled water and vacuum dried. The SEM images in **Figure** **3**a show the surface morphology evolution of the BP sheets during biomineralization. Compared to the BP sheets on day 0 with smooth surface morphology, new nanoparticles emerge the surface of the BP sheets after 2 days and the number of nanoparticles increases with biomineralization time. The nanoparticles are wrapped on the surface of BP sheets to prevent further corrosion and degradation is slowed so that it serves as a long‐term biomineralization initiator. Since phosphorus‐driven and calcium‐extracted biomineralization processes usually begins with precipitation reaction from Ca^2+^, PO_4_^3−^, and H_2_O, followed with nucleation, growth, and self‐organization of mineral crystals, such in situ biomineralization of BP may begin with the degradation of BP into PO_4_^3−^ and then extract Ca^2+^ from SBF followed with above chemical reaction and eventually formed CAP.^[^ ^52^, ^53^ ^]^

The biomineralization behavior of BP sheets under intermittent NIR illumination is assessed. As shown in Figure 3b, the nanoparticles on the BP sheets after NIR light irradiation show faster nucleation and growth than those without NIR light irradiation. After 8 days, the nanoparticles grow in quantity and size in comparison with those without NIR light irradiation and form a densely packed layer on the surface. Hence, the NIR photochemical activity of BP sheets promotes in situ biomineralization because under NIR illumination, faster enrichment of PO_4_^3−^ in local regions accelerates extraction of calcium ions from SBF and the heat generated by the photothermal effect of BP sheets also promotes nucleation and growth of the mineral crystals to accelerate biomineralization.^[^ ^21^ ^]^

The chemical composition of the nanoparticles is determined by energy dispersive X‐ray spectrometry (EDS) (Figure 3c). The distribution of O and Ca are similar to that of P indicating efficient degradation and biomineralization. XPS is employed to determine the chemical states of the BP sheets before (Figure S6, Supporting Information) and after (Figure 3d) biomineralization. The peaks at 129.5 and 130.5 eV assigned to P drop significantly, while the peak at 134.0 eV due to P*_x_*O*_y_* increases and peaks at 347.0 and 350.5 eV related to Ca^2+^ appear,^[^ ^53^ ^]^ providing additional evidence about the degradation and in situ biomineralization process on BP sheets in SBF. The P, O, and Ca concentrations after immersion for 0, 2, 4, 6, and 8 days without/with NIR light irradiation are determined by EDS (Figure S7, Supporting Information). The concentration of Ca increases gradually with time for both groups and the increase is faster with NIR light irradiation.

### 2.4 Controlled Biomineralization of BP@Hydrogel

**Figure 4:**
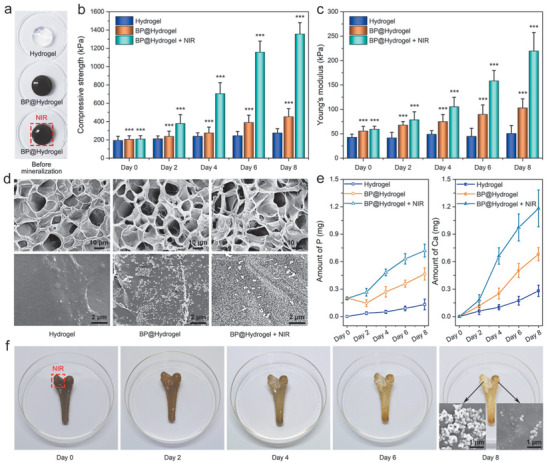
Controlled biomineralization of hydrogels arising from the NIR photochemical activity of BP: a) Photographs of hydrogels before biomineralization. b) Compressive strength and c) Young's modulus of hydrogels after biomineralization at the predetermined time intervals (0, 2, 4, 6, and 8 days). d) SEM images of the internal morphology of hydrogels after 8 days. e) Quantitative analysis of P and Ca in hydrogels after biomineralization. f) Photographs of bone‐shaped hydrogels after controlled in situ biomineralization for 0, 2, 4, 6, and 8 days with insets showing the corresponding SEM images with and without light irradiation after 8 days.

The biomineralization performance is crucial to tissue engineering especially scaffolds for cartilage and bone repair since hardened and stiffened scaffolds are necessary to provide structural support to tissues during bone regeneration.^[^ ^54^ ^]^ Based on the unique and excellent in situ biomineralization performance and NIR photochemical activity of BP sheets, NIR light controlled biomineralization of BP incorporated hydrogel (BP@Hydrogel) is demonstrated in vitro. The BP incorporated hydrogel (BP@Hydrogel) is prepared using a 4% w/v agarose aqueous solution containing 50 ppm BP sheets at 60 °C and it is then cooled rapidly to 4 °C to form the hydrogel. The photographs of the hydrogels in **Figure** **4**a reveal that the BP sheets are distributed uniformly over the agarose hydrogel and the addition of BP sheets does not destroy the structure of the agarose hydrogel.

The pure hydrogel, BP@Hydrogel, and BP@Hydrogel with NIR light irradiation are incubated in SBF for further biomineralization at 37 °C for up to 8 days. At the predetermined time intervals (0, 2, 4, 6, and 8 days), the mechanical properties of the hydrogels are determined by a dynamic mechanical analyzer. Figure S8 (Supporting Information) presents the stress versus strain curves after 8 days. The curves of the pure hydrogel and BP@Hydrogel without NIR light irradiation display a sigmoid shape characteristic of elastomeric materials with a low modulus and large deformation before fracture, whereas that of BP@Hydrogel with NIR light irradiation shows a much higher slope and bigger stress at the breaking point. The corresponding compressive strength (Figure 4b) and Young's modulus (Figure 4c) of the hydrogels at the predetermined time intervals (0, 2, 4, 6, and 8 days) are calculated from the stress–strain curves. Compared to the pure hydrogel with unobvious changes in mechanical properties during the biomineralization process, BP@Hydrogel exhibits a relatively significant mechanical enhancement, indicating that BP sheets promote in situ biomineralization of hydrogels by providing phosphorus and nucleation sites. BP@Hydrogel with NIR light irradiation has a higher compressive strength of 1350 KPa and Young's modulus of 220 KPa after 8 days and they are almost 5 times larger than those of the pure hydrogel confirming that NIR illumination accelerates biomineralization of BP@Hydrogel. Since biominerals generally possess superior mechanical properties than the synthetic pure crystals,^[^ ^55^ ^]^ BP@Hydrogel after biomineralization possess better mechanical properties (Figure S9, Supporting Information) than the sample with the same amount of CAP nanoparticles (BP&CAP@Hydrogel) due to homogeneous intermixing and combination of organic and inorganic components.

The appearance of the hydrogels before and after biomineralization is visualized after vacuum freeze‐drying (Figure S10, Supporting Information). The hydrogels show slight swelling without structural collapse during biomineralization indicating good mechanical properties. Compared to that without NIR light irradiation, BP@Hydrogel with NIR light irradiation shows a lighter color during biomineralization due to faster degradation and biomineralization. The internal morphology of these hydrogels is observed by SEM and the images after 8 days are depicted in Figure 4d. All the hydrogels have a similar porous structure further confirming no obvious structural damage during biomineralization. Compared to the pure hydrogel with few mineral particles on the wall of the inner pores, a large number of tiny mineral particles are evenly distributed on the inner pore wall of BP@Hydrogel without or with NIR illumination, indicating that the in situ biomineralization take place homogeneously on the hydrogel. In addition, more mineral particles with a dense arrangement are observed from the inner pore wall and form a mineral layer on the surface of BP@Hydrogel after NIR light irradiation. The mineral particles have a spherical structure of about 300 nm in diameter. EDS reveals the presence of phosphorus, oxygen, and calcium (Figure S11, Supporting Information) and ICP‐AES (Figure 4e) confirms the results.

Bone‐shape scaffolds are produced using the hydrogels (Figure 4f; and Figure S12, Supporting Information) by 3D printing to further investigate the controlled biomineralization process in situ. Similar spontaneous degradation and biomineralization process of BP@Hydrogel can be observed. Interestingly, the upper left part of the bone‐shape BP@Hydrogel scaffold under light irradiation by adjusting the light spot size and illumination position shows more extensive biomineralization than the upper right part without light irradiation (Figure S13, Supporting Information; and insets of Figure 4f). The results demonstrate that the NIR light‐induced photochemical activity of BP sheets can be modulated by changing the irradiation time and location. This is particularly attractive because of the high tissue penetration ability and excellent controllability in space and time of NIR light.

## 3 Conclusions

In conclusion, the photochemical activity of BP sheets arising from the formation of PO_4_^3−^ during NIR light‐controlled biomineralization in situ is systematically investigated and demonstrated. Owing to the excellent NIR absorption, BP sheets under NIR illumination exhibit much faster degradation both in moist air and solution. The chemical activity of BP sheets is enhanced by NIR light irradiation. The BP sheets not only can provide a phosphorus source and nucleation sites, but also accelerate the reaction between PO_4_^3−^ and Ca^2+^ to promote biomineralization. The BP sheets with excellent photochemical activity are applied to controlled biomineralization of hydrogels in situ and by modulating the irradiation time and location of the NIR light, the mechanical properties and biomineralization ability can be tailored. NIR light‐controlled biomineralization has immense clinical potential especially tissue engineering and the photochemical activity of BP bodes well for many other biological and biomedical applications.

## 4 Experimental Section

##### Materials

The BP crystals were purchased from Mophos and stored in a dark Ar glovebox and *N*‐methyl‐2‐pyrrolidone (NMP, 99.5%, anhydrous) was obtained from Aladdin Reagents. The SBF with an inorganic salt composition similar to human blood plasma was obtained from Qingdao Jisskang Biotechnology. Agarose was purchased from Fisher Scientific. All the chemicals used in this study were analytical reagent grade and used without further purification.

##### Synthesis of BP Flakes, BP Sheets, and BP@Hydrogel

The micro‐sized BP flakes were prepared by mechanical exfoliation from the bulk crystal using scotch tape and transferred to a Si/SiO_2_ wafer. The BP sheets were prepared by a modified liquid exfoliation technique. Briefly, the BP crystals were dispersed in NMP with an initial concentration of 1 mg mL^−1^ and ground to fine powders. The dispersion was sonicated in an ice bath for 10 h using a power of 300 W and centrifuged at 4000 rpm for 10 min. The supernatant containing the BP sheets was decanted gently and centrifuged for another 10 min at 7000 rpm. The precipitate was collected and resuspended for subsequent experiments. A certain amount of agarose powders (4 wt%) was dispersed in deionized water under mechanical stirring at 90 °C for 30 min and stored in a 4 °C refrigerator for 30 min to obtain the agarose hydrogels. BP@Hydrogel was prepared by mixing the agarose solution with BP sheets (20 ppm) by the procedures mentioned above. BP&CAP@Hydrogel was obtained by dispersing 2 mg CAP nanoparticles (≈200 nm in diameter) into the BP@Hydrogel solution and then prepared by the same method as described above. The bone‐shape hydrogels were fabricated by 3D printing using the sequential strand deposition method on a bioplotter pneumatic dispensing system (Bioscaffolder 3.1, GeSiM, Grosserkmannsdorf, Germany).

##### Characterization

The SEM images were obtained on the field‐emission SEM (NOVA NANOSEM430, FEI, Netherlands) at 5–10 kV after gold coating for 120 s (EM‐SCD500, Leica, Germany). EDS was conducted on the Oxford INCA 300 equipped on the SEM. The TEM images were acquired on the JEOL JEM‐2010 transmission electron microscope at an acceleration voltage of 200 kV and AFM was performed on an MFP‐3D‐S AFM (Asylum Research, USA) using the tapping mode in air. Raman scattering was conducted on a Horiba Jobin‐Yvon LabRam HR‐VIS high‐resolution confocal Raman microscope equipped with the 633 nm laser as the excitation source. The UV–vis–NIR absorption spectra were acquired on a Lambda25 spectrophotometer (PerkinElmer) with QS‐grade quartz cuvettes at room temperature. The concentration was determined by inductively‐coupled plasma atomic emission spectroscopy (ICP‐OES, 7000DV, PerkinElmer). XPS was carried out on the Thermo Fisher ESCALAB 250Xi XPS with an Al K_*α*_ X‐ray source. The XPS peaks were calibrated by the standard C 1 s peak at 284.8 eV according to the Thermo Scientific XPS Knowledge Base. The mechanical properties of these hydrogels (8 mm in diameter and 4 mm in height) were evaluated through a uniaxial compression test on a WDW‐05 electromechanical tester (Time Group Inc., China). The modulus was obtained by the initial (straight line) linear slope of the stress–strain curve and the compressive strength was defined as the stress at the end of the linear portion of the stress–strain curve.

##### NIR Laser Irradiation

NIR laser irradiation was performed with a fiber‐coupled continuous semiconductor diode laser (808 nm, KS‐810F‐8000, Kai Site Electronic Technology Co., Ltd. Shaanxi, China) at a power density of 1.0 W cm^−2^ for 10 min, three times daily.

##### NIR Laser‐Induced Heat Conversion

A fiber‐coupled continuous semiconductor diode laser (808 nm, KS‐810F‐8000, Kai Site Electronic Technology Co., Ltd. Shaanxi, China) was employed in the experiments. 1 mL of the sample in a 1 cm path length quartz cuvette was irradiated with the laser at a power density of 1 W cm^−2^ for 10 min. The laser spot was adjusted to cover the entire surface of the sample. Real‐time thermal imaging was performed and the maximum temperature was recorded by the Fluke Ti27 infrared thermal imaging camera (USA).

##### Degradation Performance

The freshly prepared micro‐sized BP flakes were stored in air at nearly 100% relative humidity at room temperature for different time durations and the evolution without/with NIR laser irradiation was observed by optical microscopy after exposure to moist air for 0, 2, 4, 6, and 8 days. For the degradation of BP sheets, BP sheets dispersed in water (1 mL for each, 20 ppm) were kept in closed sample vials, maintained in a horizontal shaker at 37 °C. Then the solutions were exposed to an NIR laser with a wavelength of 808 nm at a power density of 1.0 W cm^−2^ for 10 min each time for sufficient heating, naturally cool in the shaker, and then repeated three times daily for 0, 2, 4, 6, and 8 days. The concentration of phosphate anions in the supernatant was determined by the QuantiChrom Phosphate Assay Kit (BioAssay Systems) following the manufacturer's instruction.

##### In Situ Biomineralization of BP Sheets and BP@Hydrogel

The biomineralization process was carried out in the SBF solution containing similar ion concentrations as the human blood plasma. The BP sheets and BP@Hydrogel without/with NIR laser irradiation were immersed in SBF for up to 8 days with fixed at intervals at 37 °C in an incubator. After biomineralization for 0, 2, 4, 6, and 8 days, the samples were taken out, washed twice with deionized water, and vacuum freeze‐dried.

##### Statistical Analysis

All the data were presented as means ± standard deviation (SD). In order to test the significance of the observed differences between the study groups, analysis by variance (ANOVA) statistics was applied and a value of *P* < 0.05 was considered to be statistically significant.

## Conflict of Interest

The authors declare no conflict of interest.

## Supporting information

Supporting Information
