# Supplementary material for: Photochemical Activity of Black Phosphorus for Near‐Infrared Light Controlled In Situ Biomineralization
Source: Adv Sci (Weinh). 2020 May 27;7(14):2000439. doi: 10.1002/advs.202000439 (PMC7375256; doi:10.1002/advs.202000439)
Supplement: Supplementary file 1 — Supporting Information [file ADVS-7-2000439-s001.pdf]

Copyright WILEY-VCH Verlag GmbH & Co. KGaA, 69469 Weinheim, Germany, 2020.

# Supporting Information

**Photochemical Activity of Black Phosphorus for Near-Infrared Light Controlled *In Situ* Biomineralization**

*Jundong Shao, Changshun Ruan, Hanhan Xie, Paul K Chu, Xue-Feng Yu**

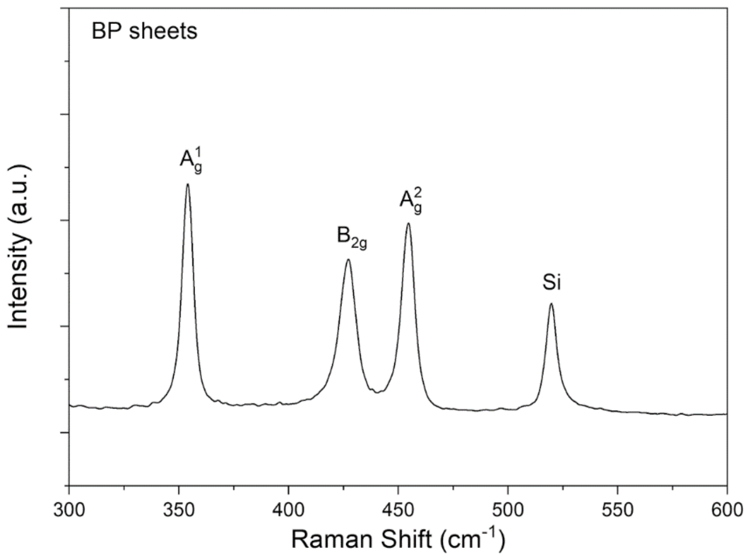


**Figure S1.** Raman spectrum of the BP sheets.

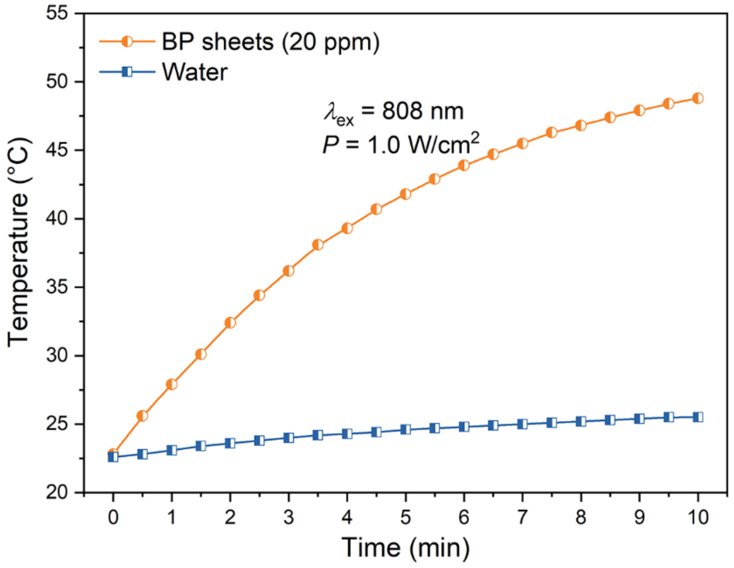


**Figure S2**. The photothermal heating curve of the BP sheets (20 ppm) irradiated with a NIR laser (808 nm, 1.0 W/cm$^2$) for 10 min.

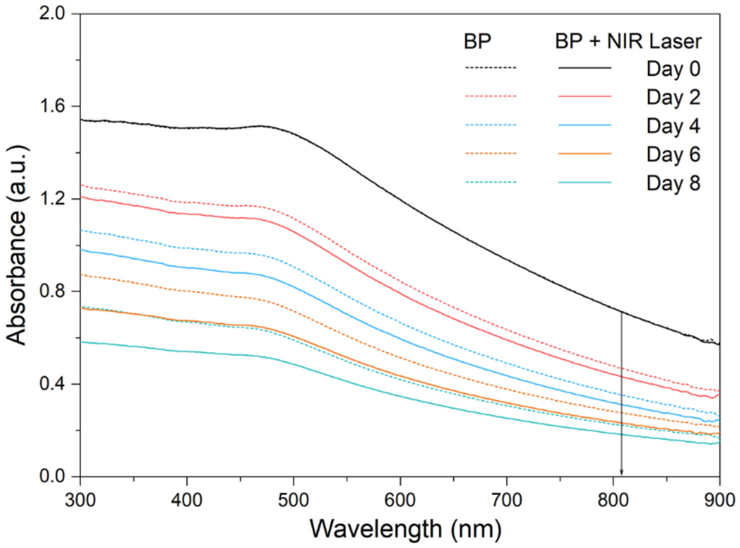


**Figure S3.** Absorption spectra of the aqueous dispersions of BP sheets without or with NIR light irradiation exposed to air for different periods of time.

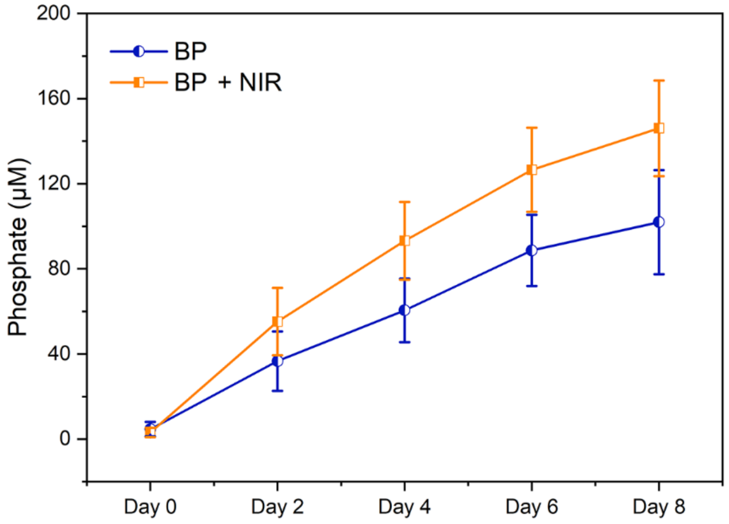


**Figure S4.** The concentration of phosphate anions after degradation of BP sheets without/with NIR laser irradiation for 0, 2, 4, 6, and 8 days.

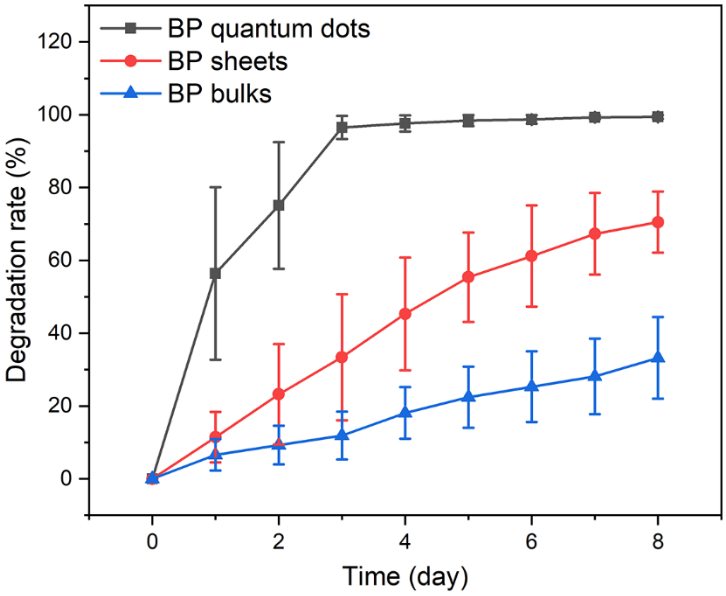


**Figure S5**. The degradation rate of BP quantum dots, BP sheets, and BP bulks in SBF for 8 days.

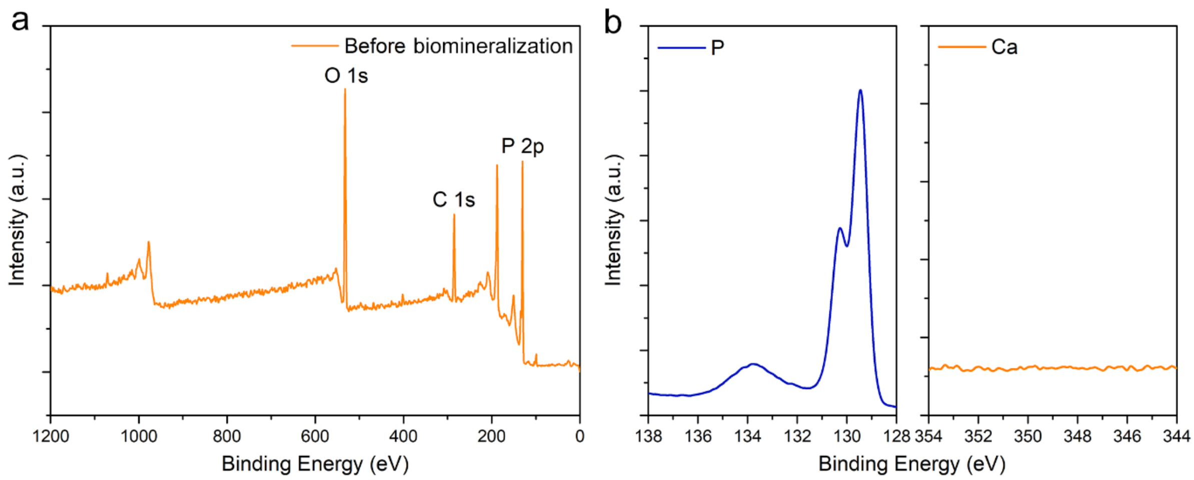


**Figure S6.** XPS spectra of BP sheets before biomineralization

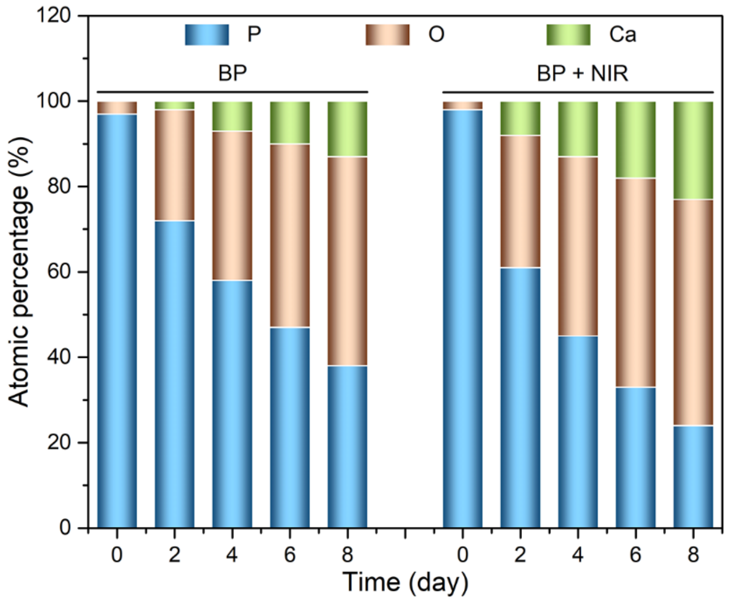


**Figure S7.** EDS analysis of the BP sheets without/with NIR laser irradiation after biomineralization for different periods of time.

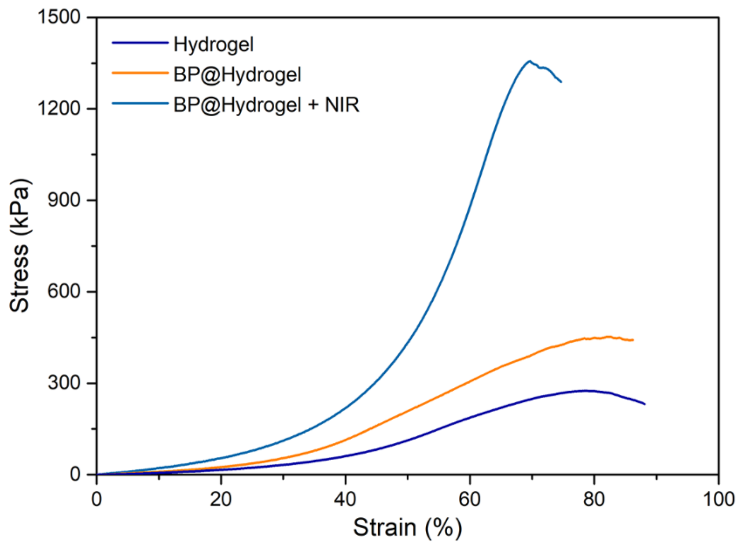


**Figure S8.** Stress-strain curves of the hydrogels after biomineralization for 8 days.

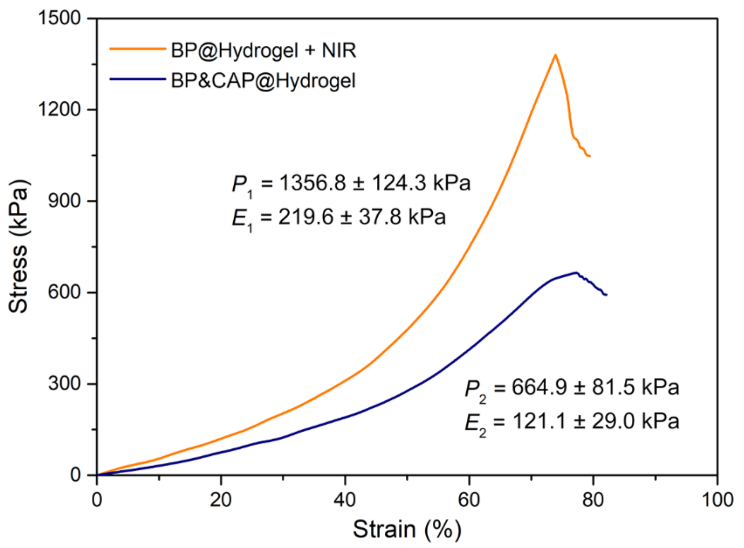


**Figure S9.** Stress-strain curves of BP@Hydrogel with NIR laser irradiation after biomineralization compared to that mixed with the same amount of CAP nanoparticles.

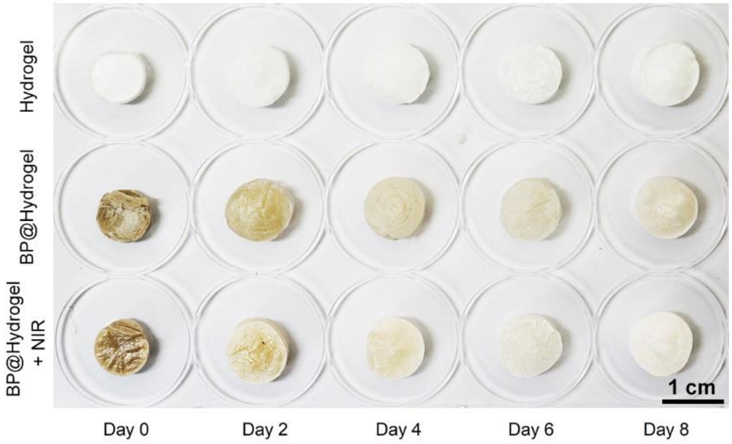


**Figure S10.** Photographs of the hydrogels after biomineralization for different periods of time.

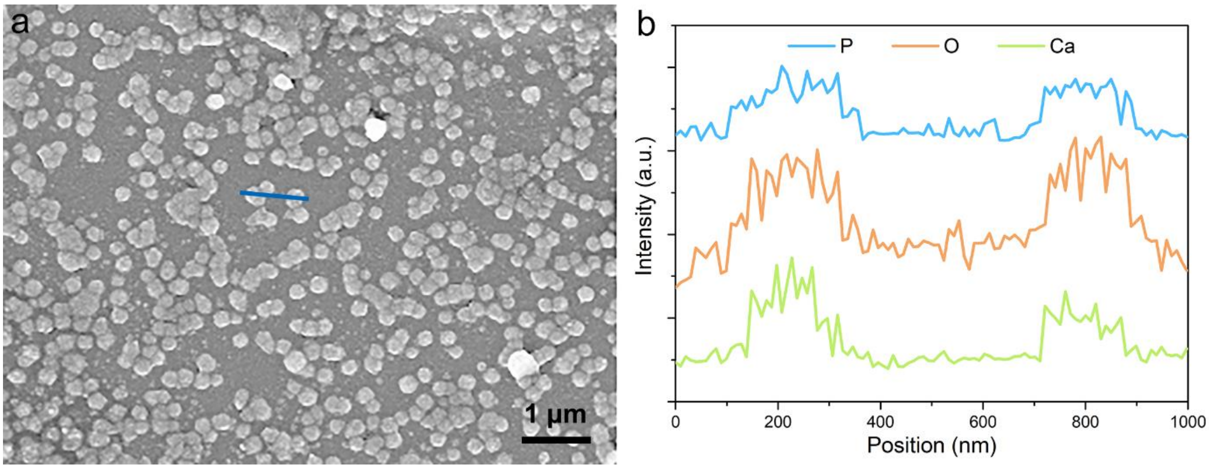


**Figure S11.** EDS analysis of the mineral particles in BP@Hydrogel after NIR laser irradiation and biomineralization.

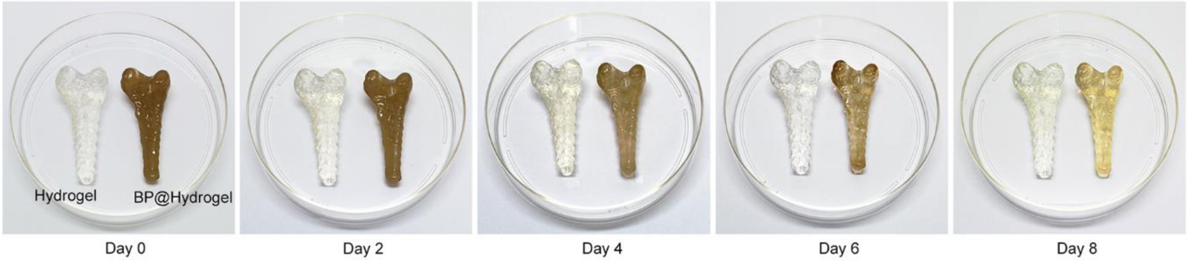


**Figure S12.** Photographs of the pure hydrogel and BP@Hydrogel after biomineralization for different periods of time.

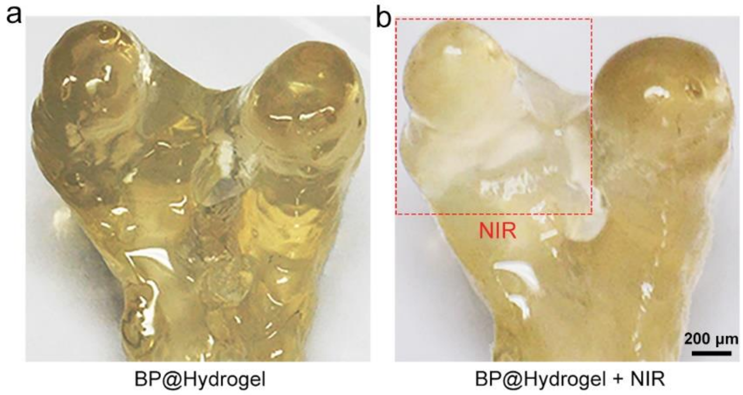


**Figure S13.** Enlarged photographs of the bone-shape BP@Hydrogel scaffolds without/with NIR laser irradiation after biomineralization.
